# PB.8. Ultrasound-guided excision of fibroadenomas: 9 years' experience in a British breast unit

**DOI:** 10.1186/bcr3716

**Published:** 2014-11-03

**Authors:** P Hamilton, A Sally, CM Lee, A Redman, S Sharma, J Westgarth, A Leaver

**Affiliations:** 1 Gateshead Hospitals NHS Trust, Gateshead, UK

## Introduction

Fibroadenomas are benign breast tumours made up of glandular and fibrous tissue. Despite their benign pathology, factors such as size, discomfort, altered breast shape and patient choice sometimes lead to excision. Traditionally, open excision biopsy has been the method of choice for fibroadenoma removal but, since 2005, our breast unit has offered patients minimally invasive ultrasound-guided complete fibroadenoma excision using vacuum-assisted core biopsy (VACB). We present an audit of our practice and patient outcomes regarding VACB excision of fibroadenomas.

## Methods

Retrospective audit of all patients that underwent ultrasound-guided VACB excision of fibroadenoma or fibroadenomata (as defined by final excision pathology) in our breast unit between January 2005 and June 2014. The authors obtained data from ultrasound images, radiology reports, pathology records and patient notes. They then performed descriptive statistics.

## Results

Eighty-three female symptomatic patients underwent ultrasound-guided VACB fibroadenoma excision as an outpatient. Lesions ranged from 7 to 44 mm in the longest axis, and from five to 180 cores were taken. Two fibroadenomas recurred (2.4%, 2/83): one at 36 months that underwent further VACB excision and one at 10 months that opted for open surgical excision. Three patients developed haematoma. Three patients had incomplete excision noted at the first VACB excision attempt, two of which were completely removed at a second sitting.

## Conclusion

Fibroadenoma excision using ultrasound-guided VACB in an outpatient setting is an effective alternative to open excision, and is associated with a low rate of recurrence and morbidity.

